# The Transcription Factor E4BP4 Is Not Required for Extramedullary Pathways of NK Cell Development

**DOI:** 10.4049/jimmunol.1302765

**Published:** 2014-02-17

**Authors:** Stefania Crotta, Annita Gkioka, Victoria Male, João H. Duarte, Sophia Davidson, Ilaria Nisoli, Hugh J. M. Brady, Andreas Wack

**Affiliations:** * Division of Immunoregulation, Medical Research Council National Institute for Medical Research, London NW7 1AA, United Kingdom; and; † Department of Life Sciences, Imperial College, London SW7 2AZ, United Kingdom

## Abstract

NK cells contribute to antitumor and antiviral immunosurveillance. Their development in the bone marrow (BM) requires the transcription factor E4BP4/NFIL3, but requirements in other organs are less well defined. In this study, we show that CD3^−^NK1.1^+^NKp46^+^CD122^+^ NK cells of immature phenotype and expressing low eomesodermin levels are found in thymus, spleen, and liver of E4BP4-deficient mice, whereas numbers of mature, eomesodermin^high^ conventional NK cells are drastically reduced. E4BP4-deficient CD44^+^CD25^−^ double-negative 1 thymocytes efficiently develop in vitro into NK cells with kinetics, phenotype, and functionality similar to wild-type controls, whereas no NK cells develop from E4BP4-deficient BM precursors. In E4BP4/Rag-1 double-deficient (DKO) mice, NK cells resembling those in Rag-1–deficient controls are found in similar numbers in the thymus and liver. However, NK precursors are reduced in DKO BM, and no NK cells develop from DKO BM progenitors in vitro. DKO thymocyte precursors readily develop into NK cells, but DKO BM transfers into nude recipients and NK cells in E4BP4/Rag-1/IL-7 triple-KO mice indicated thymus-independent NK cell development. In the presence of T cells or E4BP4-sufficient NK cells, DKO NK cells have a selective disadvantage, and thymic and hepatic DKO NK cells show reduced survival when adoptively transferred into lymphopenic hosts. This correlates with higher apoptosis rates and lower responsiveness to IL-15 in vitro. In conclusion, we demonstrate E4BP4-independent development of NK cells of immature phenotype, reduced fitness, short *t*_1/2_, and potential extramedullary origin. Our data identify E4BP4-independent NK cell developmental pathways and a role for E4BP4 in NK cell homeostasis.

## Introduction

Natural killer cells are central to innate immune defenses and patrol the organism to recognize and eliminate virus-infected, stressed, or transformed cells (1). NK cells are activated by cell-contact–dependent or cytokine-mediated stimuli (2–4), and their response includes cytotoxic function and production of IFN-γ and proinflammatory cytokines that impact on subsequent adaptive immune responses (5).

The bone marrow (BM) is the best-characterized site of conventional NK (cNK) cell development, where common lymphoid progenitors (CLPs) (6) give rise to NK cells in an IL-15–dependent process (7–10). The developing cells pass through a NK-committed precursor stage (11) with the phenotype Lin^−^CD27^+^2B4^+^CD127^+^Flt3^−^. This population can be further subdivided into pre–NK cell precursors (NKP), which do not express the IL-15R β-chain (CD122), and refined NKP, which are CD122^+^ (12, 13). The later steps of NK cell maturation follow a well-defined sequence of phenotypic changes (14–16).

Apart from the intramedullary development of cNK cells, there are also distinct extramedullary NK cell populations resident in peripheral sites, such as liver, gut, or peritoneum, which often express distinct combinations of surface markers (15, 17, 18). For instance, the liver contains a population of NK cells with an immature DX5^−^TRAIL^high^ phenotype. These stable differences in marker expression, together with the identification of peripheral precursors with NK cell potential, support the possibility of extramedullary NK cell generation (19). However, the control of NK cell differentiation in extramedullary sites is still poorly understood.

A unique population of NK cells is present in the thymus (20). Unlike BM NK cells, they express the IL-7R (CD127) and rely upon IL-7 for their development. Thymic NK cells express the maturation marker CD11b and some Ly49 receptors at lower levels than that of splenic NK cells, suggesting that they are comparatively immature (21). Although the pathway by which thymic NK cells develop has not yet been described in detail, they may originate from double-negative (DN)1 and DN2 thymocytes, which retain both NK and T cell potential (22–24). In support of this, sorted DN1 thymocytes differentiate in vitro and in vivo into cells that more closely resemble thymic, not splenic, NK cells (25).

Much work is now directed toward identifying the transcription factors required for NK cell development (26). Id2 (27, 28), Ets1 (29), T-bet, and eomesodermin (Eomes) (30) were all shown to be essential for the production of normal numbers of mature NK cells in the BM. In particular, T-bet and Eomes show high sequence similarity and have overlapping functions in NK cells (30).

Less is known about the transcriptional requirements of extramedullary NK cells. In the liver, the DX5^−^TRAIL^+^ subset of NK cells mentioned above can develop in the absence of Eomes (30). The production of thymic NK cells is critically dependent upon GATA-3 (20), whereas BM NK cells can develop in the absence of this transcription factor, despite having defects in IFN-γ production and migration to the liver (31). Therefore, the transcription factors required for intramedullary and extramedullary development of NK cells differ (32).

The transcription factor E4BP4/NFIL3 is a basic leucine zipper transcription factor that is implicated in a number of immune processes (33). It is absolutely required for the development of cNK cells in the BM, as E4bp4-deficient mice have strongly reduced numbers of mature NK cells in BM, spleen, liver, and lymph nodes (34, 35). However, the effect of E4BP4 deficiency on NK subsets in other organs has not yet been explored.

In this study, we tested the requirement for E4BP4 in NK cell development at medullary and extramedullary sites and define in vitro and in vivo conditions of E4BP4-independent NK cell development. Our data suggest that NK cells can develop independently of E4BP4, possibly through an extramedullary pathway, resulting in peripheral NK cells with reduced fitness.

## Materials and Methods

### Mice

Wild-type (wt), IL-7–deficient, E4BP4-deficient (34), and Rag-1–deficient (36) mice on the C57BL/6 background were bred and intercrossed at Medical Research Council– National Institute for Medical Research under specific pathogen-free conditions according to protocols approved by the United Kingdom Home Office and the local ethics committee.

### Influenza infection

Rag or double-deficient (DKO) mice were infected with 800 tissue culture-infective doses_50_ of X31 (a H3N2 reassortant with the backbone of A/PR/8/34) grown in Madin-Darby canine kidney cells. Single-cell suspensions were prepared from day 3 and day 6 infected lungs, and NK cells were either identified ex vivo by flow cytometry, or lung lymphocytes were stimulated for 4 h using phorbol dibutyrate/ionomycin in the presence of brefeldin A and stained for IFN-γ and NK cell markers.

### Flow cytometry

Spleens and thymi were passed through 70-μm cell strainers and washed with FACS buffer (10% BSA in PBS azide). RBCs were lysed using ammonium chloride. Livers or lungs were first homogenized using gentleMACS (Miltenyi Biotec), as per manufacturer’s instructions, and passed through a 70-μm strainer. After centrifugation, liver cells were resuspended in 40% Percoll and overlaid on 60% Percoll (GE Healthcare). Lymphocytes were then isolated at the interphase after centrifugation (30 min at 900 × *g*).

Cells were preincubated with anti-FcγRIII/II in FACS buffer prior to a 30-min incubation with one or more of the following fluorochrome-labeled Abs (Cambridge Bioscience, unless otherwise stated): anti-CD45, anti-CD45.1, anti-CD45.2, anti-CD49b (DX5), anti-NKp46, anti-Ly49G2, anti-CD122, anti-NKp46, anti-NK1.1, anti-Ly49D, anti-Ly49A/D, anti-Ly49C/I/F/H, anti-CD11b, anti-CD127, anti-Eomes, anti–T-bet, anti-CD3, anti–IL-15Rα, anti-CD4 (BD Biosciences), and anti-CD8 (BD Biosciences). NKP stains were performed using mAbs against 2B4 (R&D Systems) and CD27, CD127, CD117, CD122, Flt3, and Sca-1 (all from eBioscience). Cells were then stained with 7-aminoactinomycin D, TOPRO-3, or Aqua (Invitrogen) to exclude dead cells. Data were acquired using a LSR II or Canto (BD Biosciences) using DIVA software and analyzed using FlowJo software (Tree Star). The lineage mixture for sorting lin^−^ cells contained the biotin- or PE-conjugated Abs anti-B220, anti-CD3, anti-CD4, anti-CD8, anti-CD11b, anti-CD11c, anti-CD19, anti–GR-1, anti-NK1.1, and anti–TER-119. Binding of biotinylated Abs was visualized using allophycocyanin-Cy7–conjugated streptavidin.

### Cell culture

For bulk culture of DN thymocytes or BM precursors, sorted thymic DN (live lin^−^CD4^−^CD8^−^CD3^−^NK1.1^−^CD122^−^) cells or BM precursors (live CD11b^−^CD11c^−^CD19^−^Ter119^−^Ly6G^−^CD8α^−^CD3ε^−^CD45R^−^NK1.1^−^) were seeded at 3000 cells/well on γ-irradiated GFP-transfected OP9 or OP9-DL1 (24) stromal cells in IMDM containing 20% FBS (Stem Cell Technologies), 50 μM 2-ME, l-glutamine, and pen/strep supplemented with IL-7 (5 ng/ml), stem cell factor (SCF; 5 ng/ml), Flt3L (5 ng/ml), and IL-15 (20 ng/ml) (25). Cells received fresh media every 4 d and were cultured for 19 d in total. Alternatively, BM precursors were cultured first with SCF (100 ng/ml), Flt3L (10 ng/ml), and IL-7 (10 ng/ml) for 5 d and subsequently on OP9 cells with IL-15 (30 ng/ml) for 7 d, as described in Gascoyne et al. (34).

### Limiting dilution analysis

Sorted total DN thymocytes (live lin^−^CD122^−^NK1.1^−^CD4^−^CD8^−^CD3^−^) or the subsets DN1 (CD44^+^CD25^−^c-kit^+^), DN2 (CD44^+^CD25^+^) or DN3 (CD44^−^CD25^+^), or double-positive (DP; live NK1.1^−^CD4^+^CD8^+^) thymocytes, were cultured, as described above, with input cell numbers per well varying from 30 to 3000 cells. After 14 d of culture, cells from each well were separately stained and analyzed by FACS. Wells containing >100 CD3^−^NK1.1^+^NKp46^+^ NK cells were counted as positive. The fractions of positive and negative wells were then used to calculate the NK precursor frequency within each input population, as described by Hu and Smyth (37).

### Cytokine-induced IFN-γ production

In vitro generated NK cells rested for 36 h without OP-9 in IL-15 (10 ng/ml), or freshly obtained splenic NK cells, were cultured in IL-12 (10 ng/ml) or IL-18 (50 ng/ml) alone or in combination. After 1 h of culture, brefeldin A (2.5 μg/ml) was added, and, after 16 h of culture, cells were fixed and permeabilized (Cytofix/Cytoperm; BD Biosciences) and IFN-γ was detected by intracellular staining.

### In vitro NK cell function

For stimulation with plate-bound Abs, ELISA plates were coated with purified Abs against NK1.1 (PK136, 10 μg/ml) and NKp46 (29A1.4, 5μg/ml) overnight at 4°C. Plates were then washed with PBS and seeded with enriched NK cells from spleen. When appropriate, anti-CD107a Ab (BD Biosciences) was added to the culture at the initiation of the incubation period. Brefeldin A (10 μg/ml) and Golgi-Stop (0.7 μl/10^6^ cells; BD Biosciences) were added 2 h after the culture started. Cells were then assessed by flow cytometry for cell surface markers and intracellular IFN-γ.

For in vitro cytotoxicity assay, enriched NK cells were incubated with YAC-1 target cells in triplicate. Supernatants were harvested after 5 h and analyzed for specific lactate dehydrogenase release using the Cytotox96 nonradioactive cytotoxicity assay (Promega).

For the analysis of cell division, splenocytes were labeled for 8 min with 1 μM CFSE (Molecular Probe), washed with FBS-containing medium, and cultured for 3 d in vitro in 24-well plates (1 × 10^6^ cells/well) in the presence or absence of murine rIL-15 (PeproTech; 50 ng/ml).

### 5-ethynyl-2′-deoxyuridine in vivo proliferation assay

Mice received injections of 1 mg 5-ethynyl-2′-deoxyuridine (EdU) once or daily for 3 d and were analyzed 12 h after last injection. Single-cell suspensions of thymus, spleen, liver, and BM were stained for surface expression of NK1.1, DX5, CD11b, and CD122 and fixed in 4% formaldehyde (Sigma-Aldrich); intranuclear labeling of EdU-containing DNA was performed according to the manufacturer’s instructions (Invitrogen). The Live/Dead Aqua Stain kit (Life Technologies) was used for dead cell exclusion.

### BM chimeras

Rag-1^−/−^ or E4BP4/Rag-1^−/−^ (B6) BM cells were injected i.v. into sublethally irradiated nude (BALB/c) or BALB/c wt control mice. Organs of recipients were analyzed 8 wk or 8 mo after transplant, and donor NK cells were identified by expression of H-2b MHC and of NK1.1.

For mixed BM chimeras, E4BP4/Rag-1^−^**^/^**^−^ (CD45.2) BM cells (95%) were injected i.v. into sublethally irradiated Rag1^−/−^ mice, together with either Rag2^−/−^CD45.1^+^ or C57BL/6 CD45.1^+^ (5%) BM cells. Chimeric mice were analyzed 8 wk posttransplantation.

### Adoptive transfer studies

Nonirradiated Rag-2^−/−^IL2rg^−/−^ mice were injected i.v. with the following: 10^6^ thymocytes from Rag-1^−/−^ mice (7.6% CD3^−^, NK1.1^+^, NKp46^+^ CD122^+^ NK cells) or E4BP4/Rag1^−^**^/^**^−^ (4.9% CD3^−^, NK1.1^+^, NKp46^+^ CD122^+^ NK cells); 10^6^ purified hepatic lymphocytes from Rag-1^−/−^ mice (36.5% CD3-NK1.1^+^NKp46^+^CD122^+^ NK cells) or E4BP4^−/−^Rag-1^−^**^/^**^−^ (21% CD3^−^NK1.1^+^NKp46^+^CD122^+^ NK cells). All mice were analyzed 3 wk posttransfer.

Transfer of CD4 T cells into Rag1^−/−^ or E4BP4/Rag1^−^**^/^**^−^ mice was performed, as follows: 10^6^ CD4 T cells were MACS purified according to manufacturer’s instructions (Miltenyi Biotec) from CD45.1 B6 splenocytes and injected i.v. into CD45.1-negative recipients. Mice were analyzed 4 wk after transfer for endogenous NK cell absolute numbers in thymus, spleen, liver, and BM, as described above.

## Results

### E4BP4 deficiency affects NK cells in the spleen more than in thymus and liver

**FIGURE 1.**
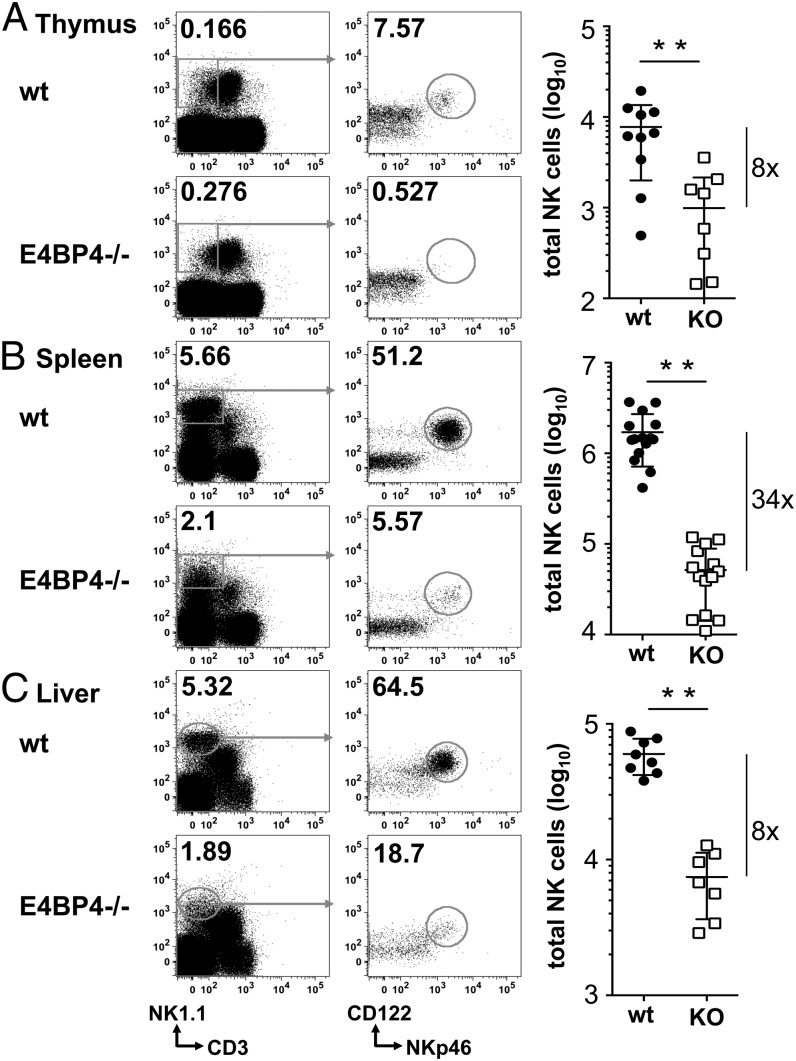
NK frequencies and numbers are reduced in E4BP4-deficient mice. The wt and E4BP4^−/−^ thymi (**A**), spleens (**B**), and livers (**C**) were analyzed for the presence of CD3^−^NK1.1^+^NKp46^+^CD122^+^ NK cells. Absolute numbers of total CD3^−^NK1.1^+^NKp46^+^CD122 NK cells were then calculated based on the gating strategy shown. Bars in the graph show means ± SD of replicates (*n* = 8–14); the fold reduction is indicated to the *right*. Asterisks indicate statistically significant differences (unpaired *t* test: **p < 0.01).

It was previously reported that the transcription factor E4BP4 is required for cNK cells in BM, spleen, liver, and lymph nodes; however, development of NK cells of other origins or presence of NK cells in other organs was not studied in detail (34, 35). Therefore, we assessed NK cell numbers and phenotypes in thymi, spleens, and livers of E4BP4-deficient mice. Total lymphocytes were first gated on the CD3^−^NK1.1^+^ population (Fig. 1, *left panels*) and further separated from the overlapping (CD3^int^NK1.1^+^) NKT cells and other innate lymphocyte cell subsets by gating on the CD122^+^NKp46^+^ population (*middle panels*). In the absence of E4BP4, frequencies and numbers of total NK cells (CD3^−^NK1.1^+^NKp46^+^CD122^+^) were significantly reduced in all organs. Splenic NK cells that are generated mainly through medullary development from BM precursors are most strongly reduced (34-fold), in line with previous results (34, 35). In contrast, thymus and liver show a comparably milder (8-fold) reduction in total NK cell frequencies and numbers. As it was shown that thymic CD3^−^ subset can include unconventional T cells with low surface CD3 levels (38), we performed intracellular CD3 staining to confirm NK cell identity in E4BP4^−/−^ mice (Supplemental Fig. 1). Stainings for surface or icCD3 yield similar frequencies (Supplemental Fig. 1A, 1B) and absolute numbers (data not shown) of NK cells and therefore confirm that we identify bona fide NK cells. In addition, T or NKT cells do not coexpress the whole set of markers used in this study to identify NK cells.

### Lack of E4BP4 mainly affects the number of NK cells with mature phenotype

**FIGURE 2.**
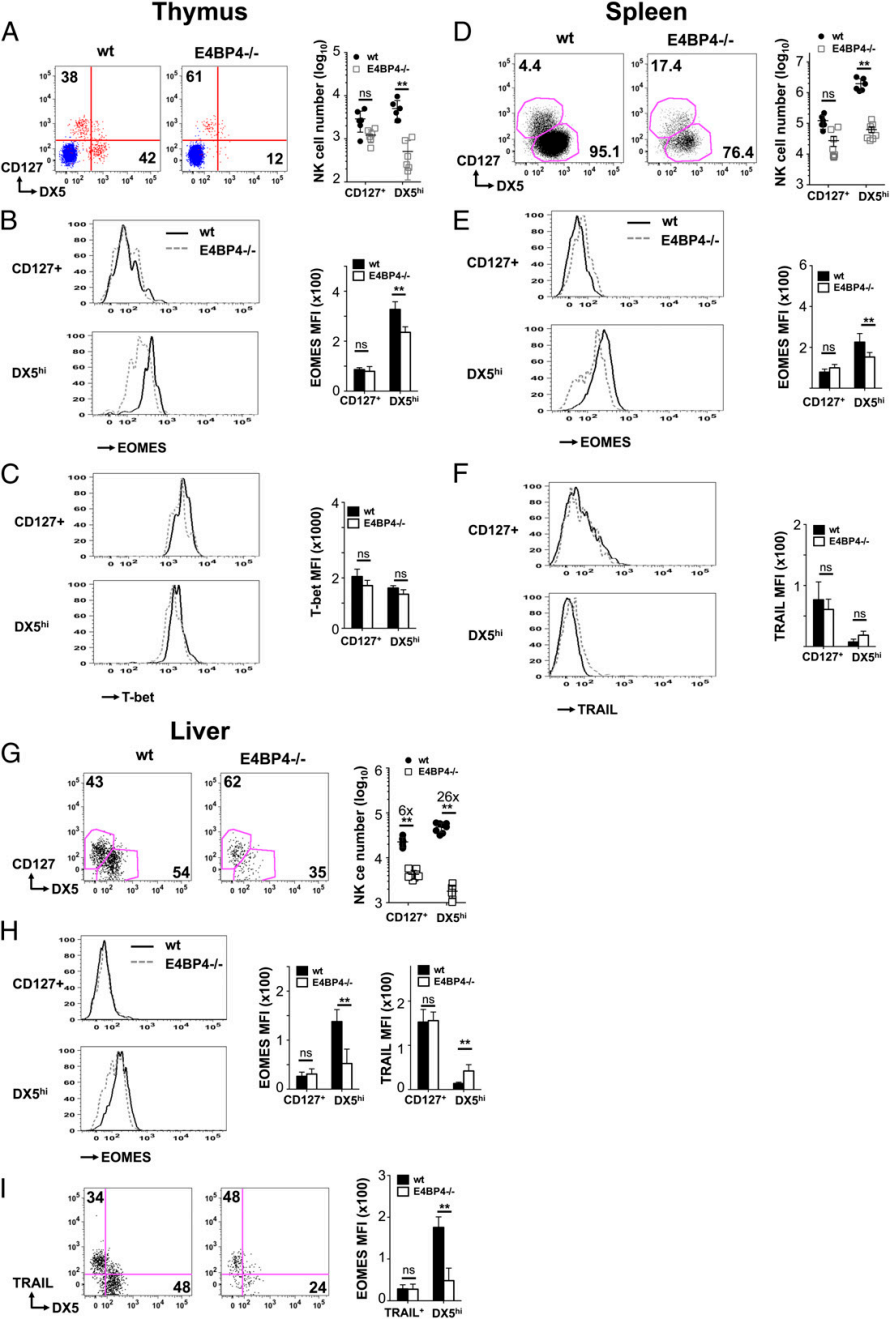
DX5^high^ Eomes^high^ NK cells are most affected by E4BP4 deficiency. Expression of CD127 and DX5 on NK cells (CD3^−^NK1.1^+^NKp46^+^CD122^+^) in wt and E4BP4^−/−^ thymus (**A**), spleen (**D**), and liver (**G**). Absolute numbers of CD127^+^ and DX5^high^ NK cells were determined, and fold reduction was indicated for the liver (G). In (A), a plot from pregated CD3^+^ T cells (in blue) was overlaid onto the NK cells (in red) to distinguish DX5^high^ and DX5^low^ NK populations from DX5^−^ T cells. (**B**, **E**, and **H**) Levels of Eomes expression were determined by intracellular staining in the CD127^+^ and the DX5^high^ NK subsets. Mean fluorescence intensity values for Eomes (B, E, and H) and for TRAIL (H) are also shown. (**C**) Expression of the transcription factor T-bet in wt and E4BP4^−/−^ thymic NK subsets. (**F**) Quantification of TRAIL expression on splenic NK subsets. (**I**) Eomes expression on TRAIL^+^ and DX5^+^ liver NK cell subsets. wt (black line); E4BP4^−/−^ (dashed line). Data are representative of at least four experiments. Error bars indicate SD. **p < 0.01, *p < 0.05. ns, not significant.

Because thymus and liver contain NK subsets of immature phenotype, some of which may be of extramedullary origin, we decided to further characterize the NK cells remaining in these organs. A separate thymic NK cell lineage was previously identified as CD127^+^ NK cells. When we further divided the total (CD4^−^CD8^−^CD3^−^NK1.1^+^NKp46^+^CD122^+^) NK cell population in the thymus on the basis of DX5 and CD127 expression, we found that the NK population separates into a CD127^+^DX5^int^ subset and DX5^high^CD127^−^ NK cells (Fig. 2A). Whereas DX5 levels differ in the NK subsets, both are positive as compared with CD3^+^ T cells in the same samples (Fig. 2A, red NK cell populations compared with overlaid blue T cell population). These two NK cell subsets are also found in spleen and liver (Fig. 2D, 2G), even though in the spleen the CD127^+^DX5^int^ subset represents only a small fraction (4.4%) of NK cells. In contrast, both in thymus and liver, this subset constitutes ∼40% of total NK cells in wt mice. When we compared Ly49 expression on these two subsets, we found Ly49D-positive NK cells only among the DX5^high^CD127^−^ subset, confirming that these cells have a more mature phenotype (Supplemental Fig. 2A–C). We then tested which of these populations is more affected by E4BP4 deficiency. Whereas the numbers of DX5^high^CD127^−^ NK cells were strongly decreased in thymus, spleen, and liver, the CD127^+^DX5^int^ subset was not significantly reduced in thymus and spleen and was 6-fold down in the liver (Fig. 2A, 2D, 2G). As DX5^high^ NK cells represent ∼95% of all NK cells in the spleen (Fig. 2D), these results explain why the greatest reduction in total numbers of NK cells is seen in this organ (Fig. 1B).

### Eomes^high^ NK cells are most affected by E4BP4 deficiency

It was previously shown that the DX5^low^ NK cells that are predominant in the liver express high levels of the surface marker TRAIL, express lower levels of the transcription factor Eomes, and constitute an immature NK subset (30, 39). We therefore assessed Eomes levels on DX5^high^CD127^−^ and CD127^+^DX5^low^ NK cell subsets in thymus, spleen, and liver. In all organs, DX5^high^CD127^−^ NK cells have significantly higher Eomes levels than CD127^+^DX5^low^ NK cells (Fig. 2B, 2E, 2H). The residual DX5^high^CD127^−^ NK cells in E4BP4-deficient mice show a significant reduction in Eomes levels, whereas the low Eomes expression in the CD127^+^DX5^int^ subsets was not affected by the presence or absence of E4BP4 (Fig. 2B, 2E, 2H). In contrast to Eomes, expression of T-bet, another transcription factor important for NK cell development, is high on both NK subsets in all organs, and its expression is unaffected by E4BP4 deficiency (Fig. 2C, Supplemental Fig. 2D, 2E). When we assessed Ly49D expression, we found that absence of E4BP4 led to a reduced frequency of Ly49D^+^ NK cells among the DX5^high^ NK cell subset (Supplemental Fig. 2A–C), which may be a reflection of the lower Eomes levels in E4BP4-deficient DX5^high^ NK cells.

TRAIL levels are low on all thymic NK cells (data not shown), whereas both splenic and liver CD127^+^DX5^int^ NK cells have higher TRAIL levels than DX5^high^CD127^−^ cells (Fig. 2F, 2I) in both wt and E4BP4^−/−^ mice. Therefore, we conclude that the CD127^+^ NK cells of thymic origin and the TRAIL^+^DX5^low^ liver NK cells described previously resemble each other in their low Eomes levels and their immature phenotype as compared with mature cNK cells of medullary origin. In conclusion, although the development of DX5^+^ cNK cells is E4BP4 dependent, the CD127^+^ and TRAIL^+^ subsets are affected by E4BP4 deficiency only regarding their cell number in the liver, with no effect of E4BP4 on any of the other parameters considered in this study, including Eomes and Ly49D expression.

### NK cells can develop from thymic precursors in vitro in the absence of E4BP4

**FIGURE 3.**
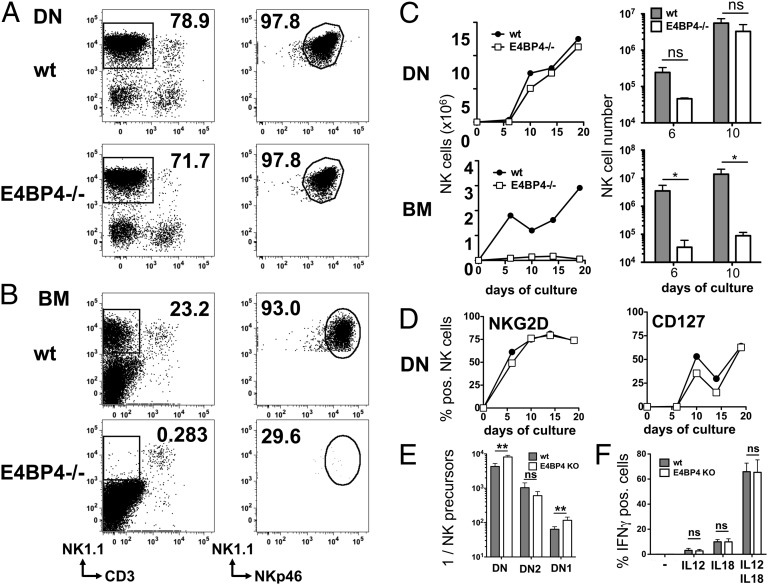
Differentiation of NK cells from thymocytes is E4BP4 independent. Differentiation of NK cells from wt and E4BP4-deficient CD4^−^CD8^−^ DN thymocytes or BM precursors. (**A**) Lin^−^CD3^−^CD4^−^CD8^−^ DN cells were sorted from wt, and E4BP4-deficient thymi or (**B**) lin^−^ precursors (CD11b^−^CD11c^−^CD19^−^Ter119^−^Ly6G^−^CD8α^−^CD3e^−^CD45R^−^NK1.1^−^CD122^−^) were sorted from BM. After 14 d of culture on OP9 feeder cells, NK cells were identified by FACS as CD3^−^CD19^−^NK1.1^+^NKp46^+^ cells (A, B). (**C**) Kinetic of NK cell development from DN or BM precursors. A single time course experiment is shown in the *left panels*; data for days 6 and 10 pooled from three experiments are shown in the *right panels*. (**D**) Kinetics of phenotypic maturation of DN-derived NK cells in vitro. (**E**) The frequency of NK precursors in total lin^−^CD3^−^CD4^−^CD8^−^ DN thymocytes or DN subsets was determined by limiting dilution analysis. Sorted cells were plated out at frequencies varying from 30 to 3000 cells/well, and frequencies of NK cell–positive and –negative wells were used to calculate precursor frequencies in the cell subsets used. Data from four experiments were pooled for this analysis. (**F**) Cytokine induced IFN-γ production by CD3^−^NKp46^+^NK1.1^+^ NK cells derived in vitro from wt or E4BP4-deficient DN thymocytes. After 14 d of culture, NK cells were incubated for 16 h with the indicated cytokines, and IFN-γ production was measured by intracellular cytokine staining. Error bars indicate the SD of replicates (unpaired *t* test: *p < 0.05, **p < 0.01). ns, not significant.

CD127^+^ thymic NK cells were described to develop along a separate thymic pathway, and we show in this study that they are less affected by E4BP4 deficiency. We therefore assessed directly whether E4BP4-deficient CD4CD8 DN thymocytes still retain a potential to differentiate into NK cells. We tested the ability of lin^−^CD3^−^CD122^−^CD4^−^CD8^−^ thymocytes to develop into NK cells under established conditions in vitro (25) and found that the absence of E4BP4 had little impact on the kinetics of NK cell numbers and phenotype as assessed over 19 d of culture (Fig. 3A, 3C, 3D). In contrast, BM precursors from E4BP4-deficient mice developed few, if any, NK cells under the same culture conditions used above (25) or in conditions that were previously described (34) and are optimized for BM precursors (Fig. 3B, 3C and data not shown). We also tested the ability of DN-derived NK cells to produce IFN-γ in response to stimulation with IL-12 and IL-18 alone and in combination and showed that IFN-γ production is independent of the presence of E4BP4 (Fig. 3F).

We then measured the NK precursor frequency in different DN subsets. We first established that, in both wt and E4BP4-deficient cells, the potential for NK cell development is present in the early DN1 (lin^−^CD3^−^CD122^−^CD4^−^CD8^−^CD44^+^CD25^−^) and DN2 (lin^−^CD3^−^CD122^−^CD4^−^CD8^−^CD44^+^CD25^+^) subsets and absent in T cell–committed DN3 (lin^−^CD3^−^CD122^−^CD4^−^CD8^−^CD44^−^CD25^+^) and in CD4^+^CD8^+^ DP thymocytes (Fig. 3E and data not shown). Further assessment of the NK precursor frequency within DN1 and DN2 thymocytes by limiting dilution analysis indicated a 2-fold reduction between wt and E4BP4-deficient DN1 thymocytes and no significant difference between the DN2 subsets (Fig. 3E). We conclude that the NK potential of E4BP4-deficient DN thymocytes is only marginally reduced.

### NK cells develop in vivo independently of E4BP4 when T cell development is blocked

**FIGURE 4.**
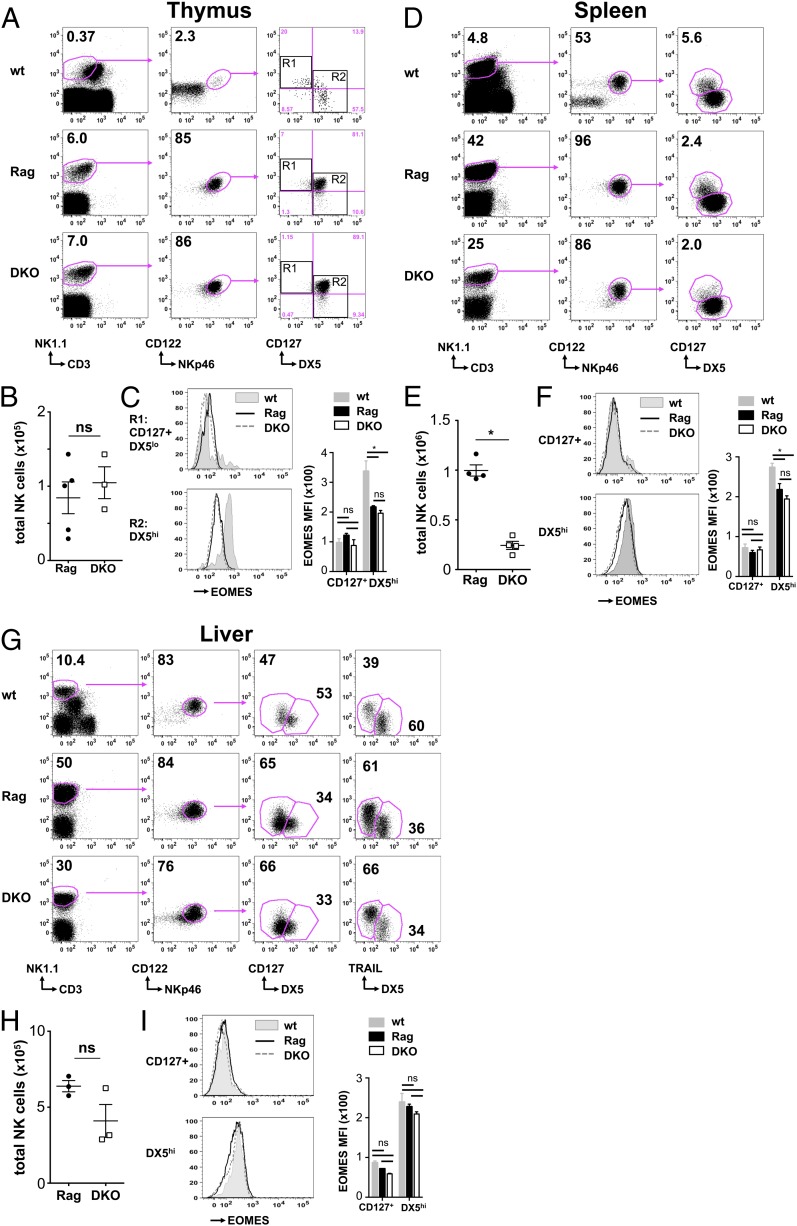
Efficient development of NK cells in E4BP4/Rag-1 DKO mice. (**A**) Total live thymocytes from Rag-1–deficient or DKO mice were analyzed by FACS for the frequency of NK cells (CD3^−^NK1.1^+^NKp46^+^122^+^). (**B**) Absolute numbers of total (CD3^−^NK1.1^+^CD122^+^NKp46^+^) NK cells in the thymus in a representative experiment of five are shown. (**C**) FACS analysis of Eomes levels in thymic CD127^+^DX5^low^ [R1 in (A)] and DX5^high^ [R2 in (A)] cells from wt (filled histogram), Rag-deficient (black line), and DKO (dashed line) mice. Overlays of single samples and quantification are shown. Total live splenocytes (**D**, **E**) and liver lymphocytes (**G**, **H**) from wt, Rag-1–deficient, or DKO mice were analyzed to determine the frequency and absolute numbers of NK cells. (**F** and **I**) FACS analysis and quantification of Eomes levels in CD127^+^DX5^low^ and CD127^−^DX5^high^ NK cells from wt (filled histogram), Rag deficient (black line), and DKO (dashed line). Error bars indicate the SD of replicates (unpaired *t* test: *p < 0.05). ns, not significant.

Given that NK cells can develop in the absence of E4BP4 but in massively reduced numbers and with an immature phenotype, we attempted to generate in vivo conditions that might facilitate the E4BP4-independent maturation of NK cells. Because DN thymocytes have NK cell potential but only few NK cells are found in the thymus, we hypothesized that Rag-1 deficiency, which blocks T and B cell development in thymus and BM, may facilitate NK cell development from thymic precursors. We therefore bred E4BP4-deficient to Rag-1–deficient (Rag) mice to obtain DKO mice. In DKO mice, NK cells (CD3^−^NK1.1^+^NKp46^+^CD122^+^) are present in the thymus at numbers that are comparable to those in the control Rag mice (Fig. 4A, 4B). These cells are negative for intracellular CD3, and therefore they do not represent unconventional T cells with low CD3 levels (Supplemental Fig. 1C, 1D). However, on the Rag background, the majority of NK cells are CD127^+^DX5^high^ and therefore differ from the thymic NK cells in wt mice (Fig. 4A).

We then compared NK cell numbers in spleen and liver of DKO versus Rag mice. The 4-fold reduction in splenic NK cell numbers (Fig. 4E) is milder compared with the 34-fold difference in NK cell numbers induced by E4BP4 deficiency in Rag-sufficient mice. In the liver, we find no significant reduction in NK cell numbers between DKO and Rag mice (Fig. 4H). There is also little change in the ratio between CD127^+^ and DX5^high^ NK cells (Fig. 4D, 4G). When we assessed the impact of E4BP4 deficiency on Eomes levels in DKO versus Rag mice, we find no change of Eomes expression between NK cell subsets from DKO and Rag mice (Fig. 4C, 4F, 4I). Like in Rag-sufficient mice, the CD127^+^DX5^low^ NK subset expresses less Eomes than DX5^high^ NK cells in all organs. Eomes levels tend to be generally lower in Rag-deficient mice than in wt mice, independently of presence or absence of E4BP4 (Fig. 4C, 4F, 4I). This difference is strongest in the thymus, less pronounced in the spleen, and absent in the liver. We conclude that, in a Rag-deficient background, lack of E4BP4 has less impact on overall NK cell numbers, on the ratio between Eomes^high^ and Eomes^low^ NK cell subsets, or on Eomes expression levels.

Because frequencies of Ly49D^+^ NK cells were lower in E4BP4^−/−^ NK cells compared with wt cells, we assessed whether this difference persists on a Rag-deficient background. All Ly49D^+^ NK cells present in the thymus are within the small CD127^−^DX5^+^ subset (Q3 in Supplemental Fig. 3A), whereas the bulk of CD127^+^DX5^high^ NK cells are Ly49D negative (Q2 in Supplemental Fig. 3A). A lower proportion of the Q3 subset is Ly49D^+^ in DKO compared with Rag control thymi (Supplemental Fig. 3B), which is similar to the NK phenotype we find in E4BP4^−/−^ thymi (Supplemental Fig. 2). In DKO thymi, Q3 NK cells also lack a proportion of panLy49^high^ cells (Supplemental Fig. 3A). In contrast, all thymic NK subsets express NKG2D, and a similar fraction expresses Ly49G2. The reduced frequencies of Ly49D^+^ NK cells seen in the thymus are also found in splenic and liver DX5^high^ NK cell subsets, indicating that E4BP4 deficiency impacts on the phenotype of these cells (Supplemental Fig. 3C, 3D). Similar to the Rag-sufficient mice, all NK cells in DKO and Rag mice were T-bet positive, and no difference in T-bet levels was found between NK subsets or between E4BP4-sufficient and -deficient mice (data not shown).

### NK cells in DKO mice are functionally normal and can develop from thymic precursors

**FIGURE 5.**
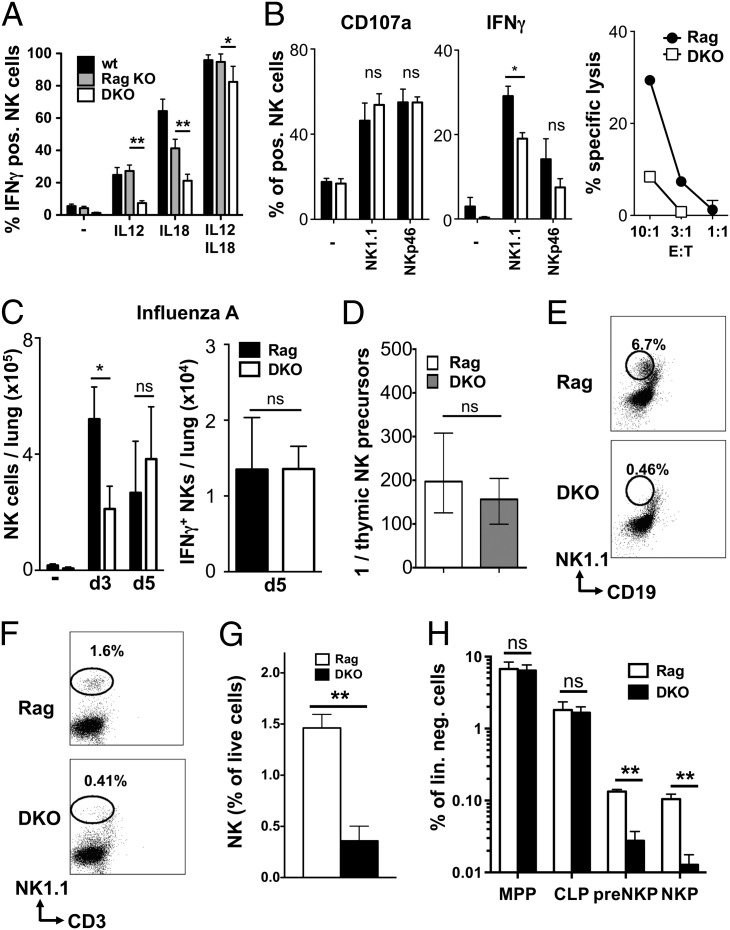
Functionality and origin of NK cells in E4BP4/Rag-1 DKO mice. (**A**) Total live splenocytes from wt, Rag-1–deficient, or DKO mice were exposed for 16 h to the indicated cytokines in the presence of brefeldin A, and IFN-γ production by NK cells was measured by intracellular cytokine staining (*n* = 3). Data shown are representative of three similar experiments. (**B**) Splenic wt and E4BP4^−/−^ NK cells were activated for 12 h with plate-bound anti-NK1.1 or anti-NKp46 Abs and evaluated for IFN-γ production and degranulation (as determined by CD107a expression). Results are representative of three independent experiments. For cytotoxicity, NK cells were mixed at the indicated ratio with YAC-1 target cells, and specific lysis was determined by net lactate dehydrogenase release from target cells. (**C**) Lungs from uninfected (−), 3-d, or 5-d influenza-infected mice of indicated genotypes were prepared for enumeration of total and IFN-γ^+^ NK cells by flow cytometry. (**D**) Determination of the frequency of NK precursors in total lin^−^CD3^−^CD4^−^CD8^−^ DN1 thymocytes by limiting dilution analysis. Sorted cells were plated at frequencies varying from 30 to 3000 cells/well, and numbers of NK cell–positive and –negative wells were used to calculate precursor frequencies. Data from three experiments were pooled for this analysis. (**E**) Lineage-depleted BM from the indicated mice was cultured first for 5 d in SCF, Flt3L, and IL-7 and then for 7 d in IL-15 with OP9 stromal cells. Cells were then harvested and examined by flow cytometry, gating by scatter and on OP9-GFP–negative cells. The frequency of NK1.1^+^CD19^−^ NK cells among live GFP^−^ cells is shown. Result representative of three independent experiments. (**F** and **G**) Frequency of mature CD3^−^NK1.1^+^ NK cells in the BM of DKO or Rag-1–deficient control mice (Rag). Individual plots (F) and mean of three mice (G) are shown. (**H**) Flow cytometric quantification of frequencies of LMPP (lin^−^Sca1^+^ckit^+^Flt3^+^CD127^−^), CLP (lin^−^Sca1^int^ckit^int^Flt3^+^CD127^+^), pre-NKP (lin^−^CD27^+^2B4^+^CD127^+^Flt3^−^CD122^−^), and refined NKP (lin^−^CD27^+^2B4^+^CD127^+^Flt3^−^CD122^+^) in BM from the indicated mice. Means of three mice are depicted. Error bars indicate SD of replicates (unpaired *t* test: *p < 0.05, **p < 0.01). ns, not significant.

In functional assays, we found lower ability of DKO NK cells to produce cytokines in response to single IL-12 or IL-18 stimulation. In contrast, there was little difference in cytokine production or degranulation in response to a combined IL-12/IL-18 stimulus or to NK1.1 or NKp46 cross-linking (Fig. 5A, 5B). Similarly, NK cell recruitment into influenza-infected lungs was only slightly delayed in DKO mice compared with Rag controls (Fig. 5C), and the recruited cells showed similar level of activation, as measured by CD69 and CD25 expression and IFN-γ production (Fig. 5C and data not shown).

To test in vitro the possible origin of NK cells in DKO mice, we tested the ability of DN1 thymic precursors and of lin^−^ BM precursors to generate NK cells. In line with our findings in Rag-sufficient mice, limiting dilution analysis indicated a similar NK precursor frequency within DKO and Rag DN1 thymocytes (Fig. 5D). In contrast, no NK cells developed from DKO BM precursors (Fig. 5E). This result parallels the lower NK cell numbers in the BM of DKO mice (Fig. 5F, 5G) and the dramatic loss of NK-committed pre-NKP and NKP populations in DKO BM (Fig. 5H, Supplemental Fig. 4). Hence, lack of E4BP4 does not affect thymic NK development in a Rag-deficient background, whereas medullary NK development is strongly reduced.

### E4BP4-independent NK cell development is not restricted to the thymus

**FIGURE 6.**
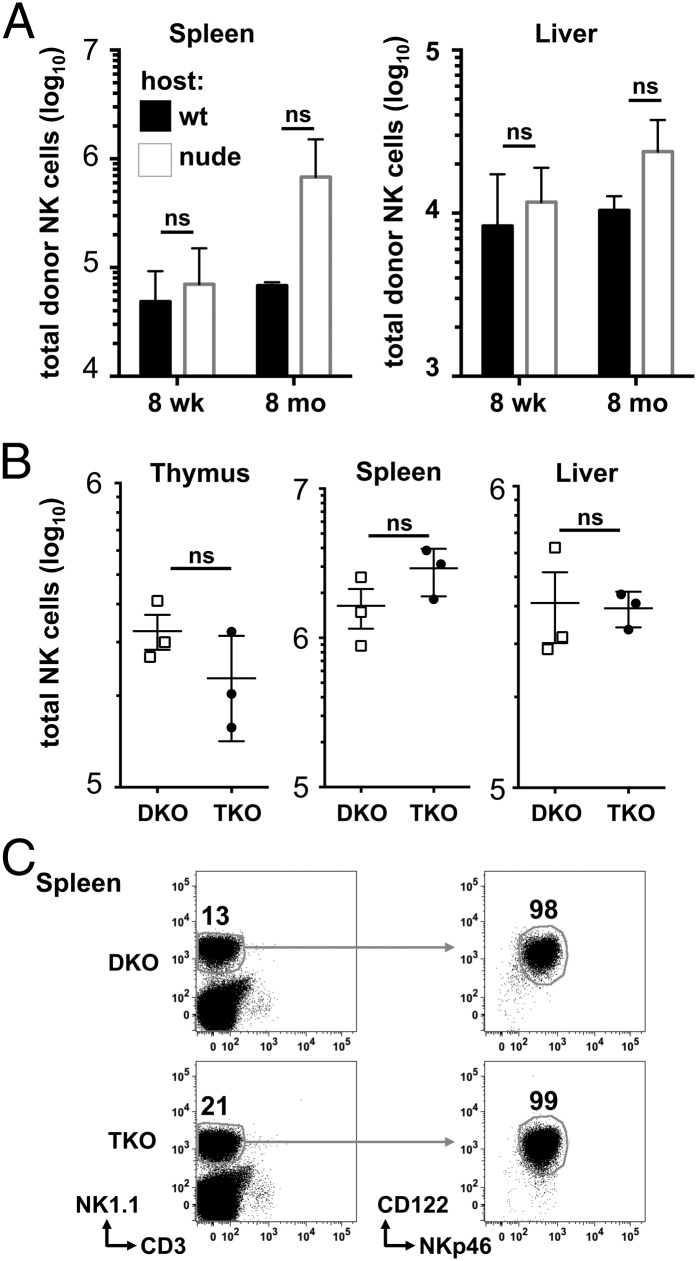
E4BP4-independent NK cell development is not restricted to the thymus. (**A**) The wt or nude recipient mice were lethally irradiated and reconstituted with donor E4BP4/Rag-1 DKO BM cells (7 × 10^6^) by i.v. injection. Chimeric mice were maintained for 8 wk or 8 mo and analyzed by FACS for the presence of NK cells (CD3^−^NK1.1^+^NKp46^+^122^+^). Absolute numbers of NK cells in the spleen and liver (A) were determined. (**B**) FACS quantification of numbers of CD3^−^NK1.1^+^NKp46^+^122^+^ NK cells in thymus, spleen, and liver of E4BP4/Rag-1 (DKO) and E4BP4/IL-7/Rag-1 (TKO) mice. (**C**) Flow cytometric plots show the characterization of the CD3^−^NK1.1^+^NKp46^+^122^+^ in the spleen of DKO and TKO animals. Numbers indicate the percentage of cells in the gates depicted.

To address in vivo whether E4BP4-independent NK cell development takes place mainly in the thymus, we generated BM chimeras by transferring DKO BM into irradiated wt controls or nude mice lacking thymic structures. We assessed reconstitution of the NK cell population after 8 wk and after 8 mo and found at both time points that presence or absence of a thymus did not impact on the numbers of donor-type NK cells in spleen and liver (Fig. 6A). Because thymic NK cells were shown to be IL-7 dependent (20), and thymocyte development is dependent on IL-7, we crossed DKO mice to IL-7–deficient mice to generate mice lacking E4BP4 and IL-7 on a Rag-1–negative background (triple deficient [TKO]). In these mice, NK cell numbers and phenotype in spleen and liver are identical to DKO controls (Fig. 6B, 6C and data not shown), indicating that E4BP4-independent NK cell development is not restricted by the lack of IL-7. Therefore, whereas the thymus may contribute to NK cell generation in DKO mice, other extramedullary origins such as the liver, or a slow and inefficient development in the BM, could contribute to the NK cell population generated in nude and TKO mice.

### E4BP4-deficient NK cells develop inefficiently and have reduced fitness

**FIGURE 7.**
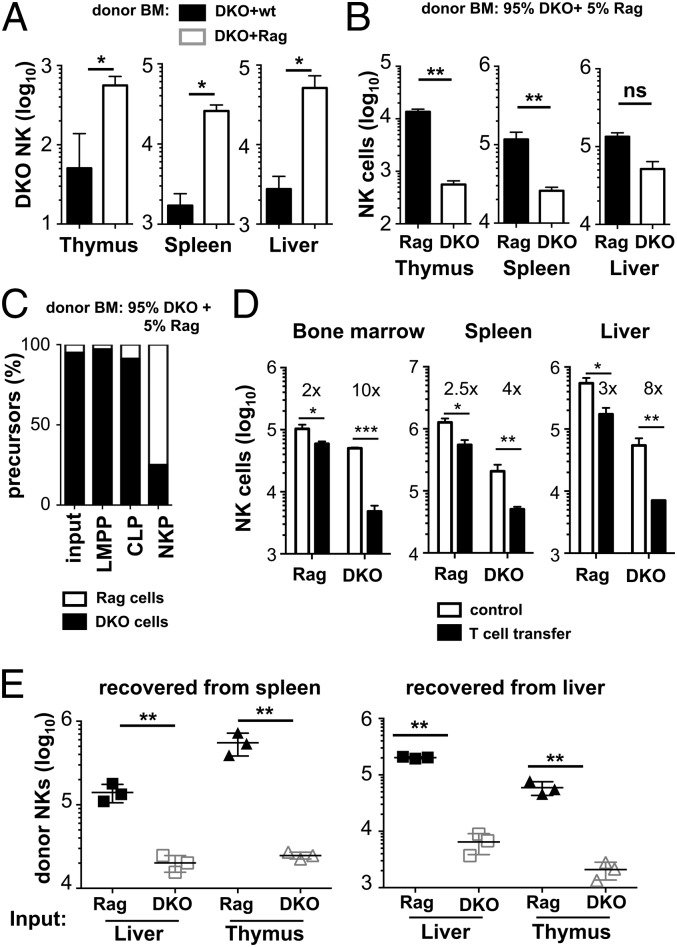
Delayed accumulation and reduced fitness of E4BP4-deficient NK cells. (**A**) Mixed BM chimeras were generated by reconstituting lethally irradiated Rag-2^−/−^CD45.1^+^ mice with a mix of either 95% DKO CD45.2^+^:5% wt CD45.1^+^ or with 95% DKO CD45.2^+^:5% Rag-2^−/−^CD45.1^+^ BM cells. The total number of CD45.2^+^ DKO NK cells in thymus, spleen, and liver was determined 8 wk posttransplantation. (**B**) Quantification of NK cells (CD3^−^NK1.1^+^NKp46^+^122^+^) in 95% DKO CD45.2^+^:5% Rag-2^−/−^CD45.1^+^ BM chimeras. Bars indicate the number of NK cells with Rag genotype as identified by CD45.1 expression or with DKO genotype expressing CD45.2. (**C**) Flow cytometric quantification of relative frequencies of DKO (CD45.2) and Rag (CD45.1) cells within the LMPP (lin^−^Sca1^+^ckit^+^Flt3^+^CD127^−^), CLP (lin^−^Sca1^int^ckit^int^ Flt3^+^CD127^+^), NKP (lin^−^CD27^+^2B4^+^CD127^+^Flt3^−^) populations in the BM of 95% DKO CD45.2^+^:5% Rag-2^−/−^CD45.1^+^ mixed BM chimeric mice. The 95% DKO: 5% Rag input is included to facilitate comparison. (**D**) Purified wt CD45.1 T cells were injected into Rag or DKO mice, and CD45.2^+^ host NK cells were quantified in the indicated organs. Fold decrease in NK cell numbers in T cell–pulsed versus nonpulsed mice is shown. (**E**) Purified hepatic or thymic lymphocytes from Rag-1 or E4BP4/Rag-1 DKO mice were adoptively transferred into IL2rg^−/−^Rag-2^−/−^ recipients and analyzed 21 d later. Total number of CD3^−^NK1.1^+^NKp46^+^122^+^ NK cells was determined in spleen and liver. Error bars indicate SD of replicates (unpaired *t* test: *p < 0.05, **p < 0.01. ***p < 0.001). ns, not significant.

The fact that E4BP4 deficiency leads to a massive NK cell reduction in Rag-sufficient mice but has almost no impact on NK cell development in Rag-deficient mice suggests that competition between different cell subsets may play a role. To address this question directly, mixed BM chimeras were generated by injecting 95% of DKO plus 5% of either wt or Rag BM cells into irradiated Rag recipients. The rationale was to understand whether the presence of T and B cells would influence the efficiency of NK cell generation. Fig. 7A shows that the presence of T and B cells in fact reduces the numbers of NK cells developing from DKO BM, confirming directly that competition with T and potentially B cells strongly impacts on the numbers of E4BP4-deficient NK cells in these mice.

We also noted in our mixed BM chimeras that, despite the 95:5 ratio of E4BP4-deficient (CD45.2^+^) to E4BP4-sufficient (CD45.1^+^) BM input, the majority of NK cells in thymus, liver, and spleen stemmed from the 5% E4BP4-sufficient BM input after 6 wk of transfer (Fig. 7B). This finding can be due to two different causes: first, it may be the result of reduced generation of E4BP4-deficient NK cells due to slow extramedullary or inefficient medullary development, whereas E4BP4-sufficient NK cells develop rapidly mainly in the BM and populate peripheral tissues. Second, there may be competition for survival or homeostatic proliferation in the periphery between E4BP4-deficient and -sufficient NK cells, with the E4BP4-sufficient NK cells prevailing. To distinguish directly between these possibilities, we first analyzed the contribution of the two genotypes to different BM precursor stages of the NK cell lineage. Although early pluripotent BM precursors (lymphoid-primed multipotent progenitors [LMPP]; Fig. 7C) are constituted of >95% of DKO precursors, closely reflecting the BM input, the contribution of DKO cells to NK-committed NKPs is dramatically reduced (Fig. 7C). This indicates that indeed the E4BP4-deficient BM input contributes little or nothing to intramedullary NK cell generation.

To understand whether mature lymphocytes compete with NK cells in the periphery, we transferred purified splenic CD45.1^+^ wt T cells into Rag or DKO mice and measured the impact on resident NK cells. Fig. 7D shows that in BM, spleen, and liver, but not in the thymus (data not shown), NK cell numbers are significantly reduced upon transfer of mature T cells. In all organs, the reduction in NK cell numbers is greater in DKO mice than in Rag mice, indicating that DKO NK cells are less able to compete with T cells than Rag NK cells, explaining the more severe NK cell phenotype in E4BP4 single-deficient mice than in DKO mice. Taken together, our data indicate that, in the absence of E4BP4, peripheral NK cells have reduced ability to survive and a reduced fitness when competing with other lymphocytes or E4BP4-sufficient NK cells.

### E4BP4-deficient NK cells are more prone to apoptosis and proliferate less well in response to IL-15

To further characterize the defects leading to reduced fitness of E4BP4-deficient NK cells in vivo, we adoptively transferred thymic or liver lymphocytes into IL-2rg^−/−^Rag-2^−/−^ recipients. Three weeks posttransfer, NK cell numbers recovered from the spleen or liver were >20-fold lower when E4BP4-deficient DKO cells were transferred as compared with Rag control cells (Fig. 7E), indicating that E4BP4 deficiency may impact on the cell’s ability to proliferate even in the absence of competition.

**FIGURE 8.**
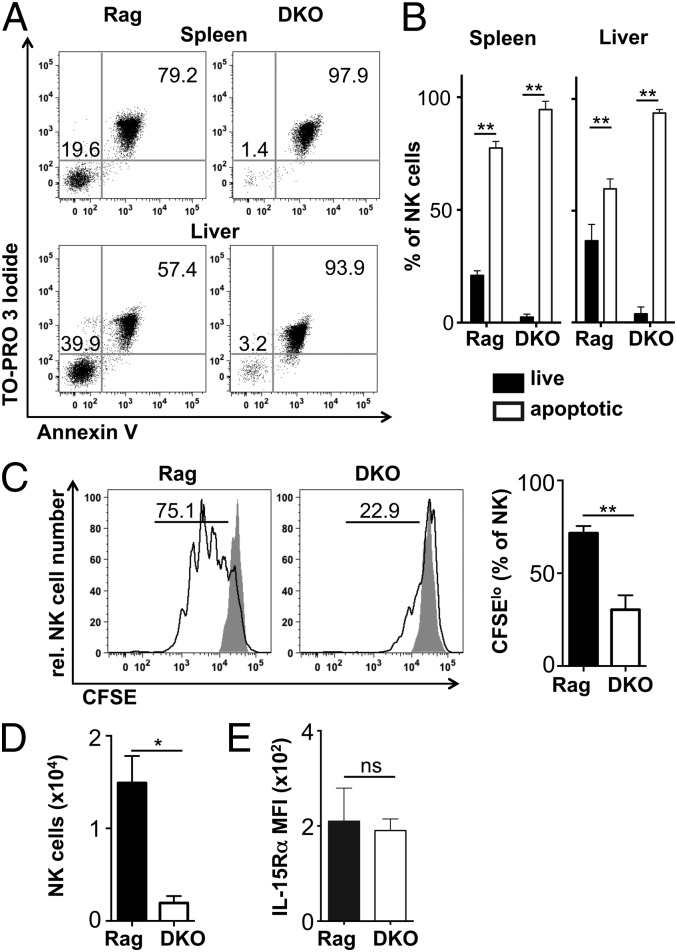
Increased apoptosis and reduced responsiveness to IL-15 of E4BP4-deficient NK cells. (**A**) Flow cytometric analysis of splenic or liver lymphocytes cultured for 12 h in complete medium. Cells were stained for NK cell markers, the DNA dye ToPro and Annexin V. Dot plots show pregated CD3^−^NK1.1^+^NKp46^+^DX5^+^CD122^+^ NK cells that are live (ToPro^−^Annexin V^−^ DN) or apoptotic and dead (ToPro^+^Annexin V^+^ DP). Cells were quantified in (**B**). (**C**) Splenocytes were CFSE loaded and incubated for 3 d in complete medium in the presence or absence of IL-15. CFSElo NK cells that have undergone at least one division are shown. (**D**) NK cell recovery at the end of the IL-15 culture period. (**E**) Splenic NK cells were stained ex vivo for IL-15Rα expression. Results are representative of three experiments. Error bars indicate SD of replicates (unpaired *t* test: *p < 0.05, **p < 0.01). ns, not significant.

To assess this, we cultured NK cells for 12 h in full medium and assessed survival and apoptosis of cells. Both splenic and hepatic NK cells from Rag mice survived better in vitro than DKO NK cells (Fig. 8A, 8B). Similar differences were found after 4 and 6 h in culture (data not shown), explaining the low NK cell recovery we had noticed in functional tests in vitro (Fig. 5A, 5B). When NK cells were incubated with IL-15 for 3 d, we found reduced cell division of DKO NK cells (Fig. 8C), resulting in lower cell recovery (Fig. 8D). The reduced response to IL-15 is not caused by lower IL-15R levels, as no differences were found between Rag and DKO NK cells by flow cytometry for the IL-15R β-chain (CD122; Fig. 4 and data not shown) or the IL-15R α-chain (Fig. 8D) or by quantitative PCR for these chains on sorted splenic NK cells (data not shown). Together, our data indicate that a higher rate of apoptosis and a reduced ability to proliferate in response to the central maintenance factor IL-15 most likely contribute to reduced NK cell fitness in E4BP4-deficient mice.

### Higher NK cell turnover in E4BP4-deficient mice

**FIGURE 9.**
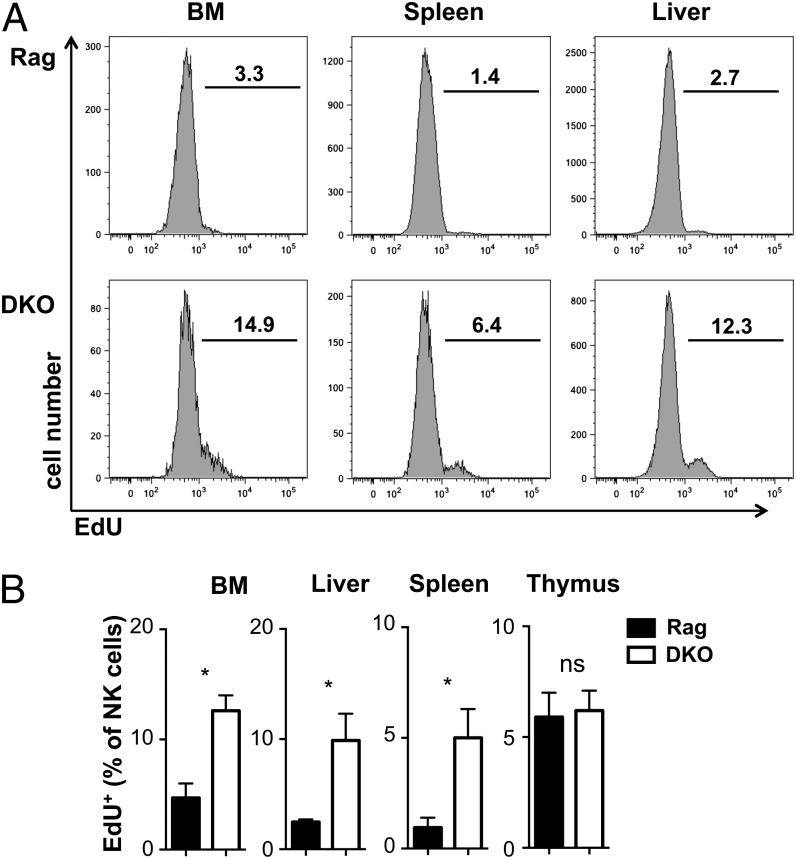
Increased turnover of NK cells in DKO mice. (**A**) Ex vivo flow cytometric analysis of EdU incorporation by NK cells after a 12-h EdU pulse in vivo. Pregated CD3^−^NK1.1^+^NKp46^+^122^+^ NK cells were identified as EdU positive, as shown in (A) and quantified (**B**). Error bars indicate SD of replicates (unpaired *t* test: *p < 0.05). ns, not significant.

Given the reduced ability of E4BP4-deficient NK cells in DKO mice to develop in the BM, the lower fitness, higher apoptosis rates, and lower responsiveness to IL-15, it could be argued that NK cell numbers are surprisingly similar in DKO versus Rag mice. To understand whether compensatory mechanisms are in action, we pulsed Rag and DKO mice with EdU for 12 h and assessed the degree of EdU incorporation, an indication of DNA synthesis and therefore proliferation within the pulse period. Fig. 9 shows that in all organs except the thymus, a higher fraction of DKO than Rag NK cells has incorporated EdU, indicating a higher degree of NK cell proliferation in DKO mice. Similar differences were found when EdU was given daily for 3 or 6 d (data not shown). This proliferation most likely serves to compensate for the reduction in NK cell development from precursors and for the lower ability of DKO NK cells to survive and compete for survival niches in the periphery.

## Discussion

During hematopoiesis, hematopoietic stem cells give rise to progressively more restricted precursor cells that include both lymphoid-restricted CLP and precursors with myeloid-restricted potential (CMP). In the mouse, restriction to the innate lymphoid lineage (including the NK lineage) is mediated by the transcription factor Id2 (12, 28), whereas several other transcription factors are required for further development along the NK lineage (40). Among these, the transcriptional activator/repressor E4BP4 is absolutely necessary for the development of cNK cells in the BM (34, 35). In this study, we reassess this requirement and define in vitro and in vivo conditions of E4BP4-independent NK cell maturation.

In E4BP4-deficient mice, the numbers of cNK cells in the thymus, spleen, and liver are strongly reduced, whereas numbers of NK cells with an immature phenotype and low Eomes expression levels are less affected. This is particularly evident in both the thymus and liver, where a high proportion of NK cells show this particular immature phenotype. We therefore claim that E4BP4 is not strictly required for the development of NK cells resident in peripheral organs.

To support this hypothesis, we demonstrate that E4BP4-deficient DN1 and DN2 thymic precursors are able to develop into NK cells in vitro with similar kinetics, precursor frequencies, phenotype, and function as wt controls. Furthermore, in Rag-1–deficient mice lacking T and B cells, NK precursor frequency and NK cell numbers in the thymus and liver are unaffected by absence of E4BP4 expression, and the phenotype and function of NK cells are similar between these Rag-1^−/−^E4BP4^−/−^ (DKO) mice and Rag-1^−/−^ (Rag) controls. These data allow us to conclude that in a T/B cell–depleted environment, NK cells have the necessary conditions to develop and expand independently of E4BP4 expression. The apparent competition for survival factors between T/B cells and NK cells is supported further by the observation that mixed BM chimeras have reduced numbers of E4BP4-deficient NK cells when T and B cells are present, or in the presence of competing E4BP4-sufficient NK cells. We could narrow the competing cell type further down to T cells, as transfer of mature splenic T cells leads to a reduction of NK cells in Rag and DKO mice. In all organs, NK cell reduction due to T cell competition is greater in DKO than in Rag mice, confirming the reduced fitness of E4BP4-deficient NK cells and explaining why E4BP4-deficient mice that are Rag sufficient show a much stronger NK cell depletion.

Despite the fact that E4BP4 expression appears to be nonessential for extramedullary NK cell development, E4BP4-deficient NK cells display reduced fitness when compared with E4BP4-sufficient counterparts, as shown by experiments in which adoptive transfer of peripheral NK cells reveals reduced ability of E4BP4-deficient cells to survive and expand in the periphery. Moreover, in in vitro experiments, E4BP4-deficient cells are more prone to apoptosis and less able to proliferate in response to IL-15. Overall, these data support the concept that NK cells with immature phenotype develop independently of E4BP4 peripheral organs and that NK cell generation from thymic precursors does not require E4BP4. However, E4BP4-deficient NK cells found in the periphery are less fit than wt NK cells and disappear rapidly in the absence of constant replenishment.

In Rag-sufficient mice, the common denominators of E4BP4-dependent NK cells are their mature phenotype, their high Eomes levels, and potentially their intramedullary development. In contrast, NK cells that do not require E4BP4 share a more immature phenotype, lower Eomes levels, and potential extramedullary origin. In general, the phenotype of tissue-resident NK cells appears more immature as compared with that of BM-derived NK cells. For instance, the liver-resident TRAIL^+^DX5^−^ subset of NK cells has a less diverse Ly49 repertoire than cNK cells (30, 39, 41). Similarly, low fractions of CD127^+^ thymic NK cells express Ly49D and other Ly49 markers, and CD11b levels are lower on thymic compared with splenic NK cells (20). In the absence of E4BP4, numbers of mature DX5^high^ NK cells are reduced in all organs assessed, and the remaining cells show a changed Ly49 repertoire, with fewer cells expressing Ly49D and other Ly49 family members, indicating that the remaining DX5^high^ NK cell populations have a more immature phenotype.

This clear difference in E4BP4 requirements between mature and immature NK cell subsets suggests that either there are distinct, E4BP4-dependent, and independent developmental pathways, or that the more immature NK cells found in E4BP4^−/−^ mice are blocked at some stage of their maturation. We favor the hypothesis of distinct pathways based on our findings that thymic, but not BM, precursors allow E4BP4-independent NK cell development in vitro. In addition, the normal number of NK cells in thymus and liver of Rag-1/E4BP4 DKO mice are in contrast to strongly reduced NK cell frequencies and NK precursor numbers in the BM of these mice, suggesting that the majority or even all NK cells we find in DKO mice are of extramedullary origin. In vivo, we do not find evidence of the thymus as the sole organ required for E4BP4-independent NK cell development, but other sites such as the liver may contribute as well. Alternatively, intramedullary development may be strongly reduced but not completely blocked in E4BP4-deficient mice, thus allowing accumulation of NK cells in specific sites over time.

NK cell numbers are similar in thymus and liver of DKO mice and Rag controls and reduced only by a factor of 4 in the spleen. In contrast, NK cell frequencies and numbers are more strongly affected by E4BP4 deficiency in Rag-sufficient mice. Adoptive transfer and BM chimera experiments show that the more severe phenotype of E4BP4 single-knockout mice is at least partly due to a reduced fitness that becomes evident in the presence of competing cells, but less so in their absence in DKO mice. The development of E4BP4-deficient NK cells in DKO mice has allowed us further examination of the NK cell deficiencies in the absence of this transcription factor. We confirm a severe block in intramedullary NK cell development and find in addition that peripheral maintenance is characterized by an overall higher turnover of the NK cell population in DKO compared with Rag mice that compensates for a reduced responsiveness to the NK survival factor IL-15 and a higher frequency of apoptosis of peripheral NK cells. In contrast, NK cell functions such as degranulation and cytokine production in response to inflammatory cytokines or receptor cross-linking in vitro and to virus infection in vivo are only mildly affected when measured by single-cell assays. Because DKO NK cell survival in vitro is compromised, we think that the reduction in cytotoxicity we observe in in vitro assays is partly, if not mainly, due to NK cell loss during this bulk assay. Together, our data point to a major role of E4BP4 in NK cell development and for maintenance in a competitive peripheral environment, whereas mature NK cell functions are largely independent of E4BP4.

A recent report of E4BP4 deletion induced postintramedullary development finds that the mature NK cell population is maintained in the absence of E4BP4, results that are largely in agreement with our findings in Rag-negative mice (42). This study did, however, not analyze NK cell turnover or IL-15 responsiveness; therefore, the NK population followed in that study may have homeostatic dynamics that are different from wt NK populations, as was the case when we compared NK cell turnover in Rag and DKO mice. At difference with our results, Firth et al. (42) find no loss of competitive fitness upon E4BP4 deletion in peripheral NK cells, which may suggest that the absence of E4BP4 during development (in our system) prevents induction of factors crucial for NK cell homeostasis in a competitive environment.

Previous studies have shown that, in the absence of E4BP4, transcription factors crucial for intramedullary NK cell development such as Id2 and Gata3 are not upregulated, leading to a developmental block (34, 35). We confirm this block and find no indication in vitro that BM cells develop into NK cells independently of E4BP4. In contrast, E4BP4-deficient NK cells can develop from DN thymocytes in vitro, and preliminary analysis showed no significant differences between wt and E4BP4-deficient NK cells in levels of Id2 or Gata3 (data not shown). This suggests that there are E4BP4-independent pathways to induce these transcription factors, ultimately leading to the establishment of an almost complete NK expression program. It was previously shown that Gata3 is important for the development of thymic NK cells (20). We suggest that NK cell development from DN thymocytes involves T cell–related transcription factors including Gata3 (43) and thereby bypasses the necessity of E4BP4 for induction of more downstream transcription factors. In contrast, BM precursors appear to be more dependent on E4BP4 and less able to use alternative pathways, circumventing the requirement of E4BP4 for development into the NK cell lineage.

Downstream transcription factors such as T-bet and Eomes have been shown to be required for NK cell identity (30), and it is possible that E4BP4 plays a role in their induction. Both cNK cells and a newly described intraepithelial innate lymphocyte cell 1 subset express high Eomes levels and are reduced in the absence of E4BP4 (34, 35, 44). This and the independent, copublished study by Seillet et al. (45) demonstrate that the naturally Eomes^low^ thymic CD127^+^DX5^low^ and liver DX5^−^TRAIL^+^ NK cell populations are less affected by E4BP4 deficiency than Eomes^high^DX5^+^CD127^−^TRAIL^−^ NK cells. The residual NK cells with mature phenotype present in E4BP4-deficient mice show lower Eomes levels than the corresponding subset in wt mice. In contrast, T-bet levels are similar in all NK cell subsets and in E4BP4-deficient and control mice. These data are consistent with a role of E4BP4 in the upregulation specifically of Eomes but not of T-bet in NK cells.

We also show that NK cells in Rag-1–deficient thymi have lower Eomes levels compared with wt cells. A similar trend is found in other organs assessed in this study. The more immature, Eomes^low^ NK subsets of CD127^+^ and TRAIL^+^ NK cells in thymus and liver are also increased in Rag-1–deficient mice. In addition, Eomes levels in NK cells do not differ significantly between DKO and Rag controls, which may help explain the lower impact of E4BP4 deficiency on NK cell development in these genetic backgrounds. If extramedullary NK cell development requires high Eomes levels in a Rag-1–sufficient context, then lack of E4BP4 and subsequent lack of Eomes upregulation will lead to a reduction in this cell population. In contrast, if low amounts of Eomes are sufficient for NK cell development and more NK cells are Eomes^low^ in the absence of Rag-1, then presence or absence of E4BP4 will be of little consequence, as seen in the DKO mice. The Rag-dependent developmental block may facilitate NK cell development either by allowing precursor commitment to the NK cell lineage or by lack of competition for thymic niches or soluble factors such as IL-7 that were shown to be crucial for thymic NK cell development (20). However, the efficient NK cell development in TKO mice shows that IL-7 is not crucial for E4BP4-independent NK cell development.

In conclusion, we demonstrate in vivo and in vitro that NK cells can develop independently of E4BP4, leading to populations with immature phenotype. We propose that separate, extramedullary developmental pathways, for instance in the thymus or the liver, may contribute to the generation of these NK cells. Our data indicate that transcription factor requirements may vary for different pathways of NK cell development. In the absence of competing lymphocyte populations, E4BP4-independent NK cell development and survival are facilitated, but resulting NK cells turn over faster as a consequence of higher apoptosis rates and lower responsiveness to IL-15. Taken together, our data suggest that E4BP4 affects not only NK cell development and phenotype but also their fitness and survival.
